# Sodium Butyrate Promotes the Differentiation of Rat Bone Marrow Mesenchymal Stem Cells to Smooth Muscle Cells through Histone Acetylation

**DOI:** 10.1371/journal.pone.0116183

**Published:** 2014-12-30

**Authors:** Jingxia Liu, Yanzhou Wang, Yuzhang Wu, Bing Ni, Zhiqing Liang

**Affiliations:** 1 Department of Gynecology and Obstetrics, Southwest Hospital, Third Military Medical University, Chongqing 400038, PR China; 2 Institutions of Immunology, PLA, Third Military Medical University, Chongqing 400038, PR China; The University of Adelaide, Australia

## Abstract

Establishing an effective method to improve stem cell differentiation is crucial in stem cell transplantation. Here we aimed to explore whether and how sodium butyrate (NaB) induces rat bone marrow mesenchymal stem cells (MSCs) to differentiate into bladder smooth muscle cells (SMCs). We found that NaB significantly suppressed MSC proliferation and promoted MSCs differentiation into SMCs, as evidenced by the enhanced expression of SMC specific genes in the MSCs. Co-culturing the MSCs with SMCs in a transwell system promoted the differentiation of MSCs into SMCs. NaB again promoted MSC differentiation in this system. Furthermore, NaB enhanced the acetylation of SMC gene-associated H3K9 and H4, and decreased the expression of HDAC2 and down-regulated the recruitment of HDAC2 to the promoter regions of SMC specific genes. Finally, we found that NaB significantly promoted MSC depolarization and increased the intracellular calcium level of MSCs upon carbachol stimulation. These results demonstrated that NaB effectively promotes MSC differentiation into SMCs, possibly by the marked inhibition of HDAC2 expression and disassociation of HDAC2 recruitment to SMC specific genes in MSCs, which further induces high levels of H3K9^ace^ and H4^ace^ and the enhanced expression of target genes, and this strategy could potentially be applied in clinical tissue engineering and cell transplantation.

## Introduction

Stem cell transplantation is an attractive option for the treatment of bladder function disorders such as stress urinary incontinence (SUI) [1]. Adult autologous muscle derived stem cell transplantation was recently used to treat SUI and has made some promising progress [2]. However, the potential application of this treatment is greatly hampered by the limited number of stem cells, as sufficient stem cells are needed to meet the requirements of cell transplantation.

Because bone marrow mesenchymal stem cells (MSCs) are easily obtained, have no ethical concerns and have little immunogenicity, these cells are considered an alternative transplantation seed [3], [4]. Although previous *in vitro* or *in vivo* experiments have demonstrated that autologous MSCs can differentiate into bladder smooth muscle cells (SMCs) [5], it is still necessary to identify novel strategies and the mechanisms underlying these strategies in order to improve the differentiation efficiency of MSCs and support the promising application of MSCs in clinical tissue and organ transplantation.

Recent studies have revealed that epigenetic regulation plays a crucial role in SMC differentiation [6]. The promoters of SMC specific genes exhibit a high level of histone modifications compared to embryonic stem cells (ESCs) [7], [8]. However, whether such histone modifications affect the differentiation of MSCs to SMCs is poorly understood.

Of the epigenetic regulation mechanisms, histone acetylation primarily promotes the expression of target genes [9]. Histone acetylation is adjusted through acetyltransferases (HATs) and histone deacetylases (HDACs) [9]. HDACs can arrest stem cell proliferation and induce cell differentiation and apoptosis [10]. Of the HDACs, HDAC1 and HDAC2 are widely expressed in the nucleus, and the levels of these proteins correlate with SMC differentiation [11], [12].

Sodium butyrate (NaB), a histone deacetylase inhibitor, has been found to play an important role in stem cell differentiation [13]. NaB promotes stem cell differentiation by suppressing HDAC activity [14], [15], and NaB can specifically induce the generation of hepatic progenitor cells from ESCs [16] and osteoblast differentiation from MSCs [17], suggesting that NaB might be an effective regulator to promote MSC differentiation into certain terminal cell types. However, whether and how NaB influences MSC differentiation into SMCs by targeting HDAC1/2 and the subsequent acetylation of target genes remains unknown.

In the present study, we found that NaB effectively promotes MSC differentiation into SMCs. NaB markedly inhibited the expression and enrichment of HDAC2 at SMC specific genes in MSCs, which further induced high levels of H3K9 and H4 acetylation and the subsequent expression of the SMC specific genes α-SMA, calponin and SM-MHC. These results provide a strategy and mechanism for promoting MSC differentiation that could potentially be applied in clinical tissue engineering and cell transplantation.

## Materials and Methods

### MSCs

Four-week-old female Sprague Dawley (SD) rats were bred and obtained from the laboratory animal center in the Third Military Medicine University. Rats were sacrificed by cervical dislocation and bone marrow MSCs were aseptically isolated from the femurs and tibias by using a 10 ml syringe to wash the marrow cavity. The harvested MSCs were cultured in DMEM/F12 medium (Invitrogen, CA, USA) with 10% FBS at 37°C with 5% CO_2_. The adherent cells were passaged at 80%–90% confluency. The third or fourth passage cells were checked for MSC markers using flow cytometry (**S1 Fig** **.**) and were negative for CD31, CD34 and CD45, but positive for CD29 expression. The identified MSCs were then co-cultured with SMCs for further analysis. This study was specifically approved by and all experiments were performed according to the guidelines set by the Laboratory Animal Welfare and Ethics Committee of the Third Military Medical University, Chongqing, China.

### Bladder SMCs

Bladders were isolated from four-week-old SD rats and digested with a 1∶1 mixture of collagenase I and II after the removal of the serosal and mucosal layers. The free cells were then collected and cultured in H-DMEM medium (Invitrogen) with 10% FBS at 37°C with 5% CO_2_. The SMCs were passaged and the third or fourth passage cells were used for co-culturing with MSCs.

### MSC-SMC co-culture

The MSCs and SMCs were co-cultured in a transwell chamber that was used according to the manufacturer’s instructions (Millipore, Billerica, MA). The SMCs were loaded in the chamber insert and the MSCs pretreated with or without NaB for 48 h were seeded in the plate well. The MSCs were then harvested at different time points for further analysis.

### Cell growth assay

MSCs were plated into 96-well plates at a concentration of 400 cells/well. The cells were pretreated with various concentrations of NaB and the cell proliferation was analyzed with the Cell Counting Kit-8 (CCK-8, Beyotime, Beijing, China) 24 h, 48 h and 72 h after the pretreatment period. Ten microliters of the CCK8 solution were added to each well, after which the plates were incubated at 37°C for 4 h. The absorbance of the samples at 450 nm was measured with a Microplate Reader (Bio-Rad, CA, USA).

### Immunofluorescence Assay

The cells were fixed directly in the plate wells with 4% formaldehyde for 10 min before being incubated with primary antibodies against α-smooth muscle actin (α-SMA) (1∶200, Abcam, San Francisco, CA, USA), calponin (1∶150, Abcam) and smooth muscle myosin heavy chain (SM-MHC) (1∶150, Abcam) at 4°C overnight. After incubation with a FITC-conjugated IgG (1∶150, Santa Cruz Biotechnology, Santa Cruz, CA, USA) secondary antibody for 30 min, the cell nuclei were stained with 4′, 6-diamidino-2-phenylindole (DAPI) for 5 min. The cells were then imaged with confocal microscopy.

### Quantitative real-time polymerase chain reaction (qPCR)

Total RNA was extracted for reverse transcription with a kit from Takara (Takara, Dalian, China). The cDNA was synthesized using a PrimeScript 1st Strand cDNA synthesis kit (Takara). The real-time PCR was performed with a quantitative real-time amplification system (MxPro-Mx3000P; Stratagene, La Jolla, CA). SYBR Green PCR Master Mix (Applied Biosystems, Foster City, CA) was used in each reaction. The relative gene expression levels were calculated by normalizing the quantified cDNA transcript level to the β-actin transcript level. The primer sequences were as follows: α-SMA: 5′-GGGCATCCACGAAACCACCTAT-3′ (forward) and 5′-CGCCGATCCAGACAGAATATTTG-3′ (reverse); calponin: 5′-CCCACAATCACCACCCACACAAC-3′ (forward) and 5′-CCTCGGCCTGATCTCCCCAAACT-3′ (reverse); SM-MHC: 5′-GAGGAGGCGGTGCAGGAGTGTAG-3′ (forward) and 5′-GGCGCTGGTGTCCTGCTCCTT-3′ (reverse) and β-actin: 5′-TGGTGGGΑΑTGGGTCΑGΑΑG-3′ (forward) and 5′-ΑCGCΑCGΑTTTCCCTCTCΑG-3′ (reverse). The annealing temperature was 60°C. Each value represents the average of at least 3 independent experiments.

### Western blot assay

The cells were washed three times with ice-cold PBS, lysed in sodium dodecyl sulfate (SDS) lysis buffer and centrifuged, and the supernatants were collected. A total of 15 µg protein was loaded and separated on a 10% polyacrylamide gel and transferred to a nitrocellulose membrane. After blocking, the membranes were incubated with the following primary antibodies: anti-α-SMA (1∶200, Abcam), anti-calponin (1∶20000, Abcam), anti-SM-MHC (1∶1000, Abcam), anti-HDAC1 (1∶1000, Millipore) and anti-HDAC2 (1∶1000, Millipore). The membranes were then developed with an enhanced chemiluminescence reagent (ECL; Amersham, Piscataway, NJ, USA). The same sample was analyzed with an anti-mouse β-actin antibody as a control for protein loading.

### ChIP-qPCR

Chromatin immunoprecipitation (ChIP) was performed using a ChIP assay kit (Millipore). In brief, 1×10^7^ cells were fixed with 1% formaldehyde for 10 min at room temperature. The cross-linked DNA was sheared to 200–500 bp in length by sonication. ChIP grade antibodies against acetyl-histone H3K9 (5 µg, Abcam), acetyl-histone H4 (5 µg, Millipore), HDAC1 (1 µg, Abcam) and HDAC2 (1 µg, Abcam) were incubated with each immunoprecipitation reaction at 4°C overnight. Normal rat IgG (5 µg, Abcam) was used as a negative control. After reversing the crosslinks, the DNA was purified and analyzed using qPCR. The ChIP-qPCR data was normalized to the normal rat IgG control in each experiment. The primer sequences were as follows: α-SMA: 5′-CCGCAGTTACAGTGATTC-3′ (sense) and 5′-GCTAAGGGCTTGAGATGA-3′ (anti-sense, 275 bp), calponin: 5′-CAGTCAGCTCCCAATACCAA-3′ (sense) and 5′-TTCCAAGCCTGTCCCATT-3′ (anti-sense, 112 bp), and SM-MHC: 5′-CAGATCCGTACAGGGCTAA-3′ and 5′-TGCCTGGGACCAATAAC-3′ (anti-sense, 179 bp). The annealing temperature was 60°C.

### Intracellular Ca^2+^imaging

Cells were cultured on a 35 mm laser confocal petri dish and loaded with Fluo-8/AM (2.5 µm; AAT Bioquest, Inc., USA) at room temperature for 1 h in the dark. After washing twice with D-Hank’s solution, the intracellular Ca^2+^ changes in these samples were recorded using a microscope system. The Fluo-8 was excited at 490 nm, and the fluorescence emission was measured at 514 nm. The cells were continuously superfused with D-Hank’s solution. Images were acquired at 2-second intervals during treatment with carbachol and high potassium. The data are expressed as F/F0, where F is the absolute florescence value and F0 is the baseline.

### Statistical analysis

We used three or four rats for each experiment by pooling and mixing the total MSCs from these rats, and distributed these cells into three parallel wells. Each experiment was repeated for three times. The data shown were the results from one of three independent experiments and presented as mean ± SD. Comparisons between two groups were made using a t-test or one-way ANOVA, and comparisons between more than two groups were made using Tukey’s Post-hoc analysis. The data were analyzed using GraphPad Prism 5.0 (GraphPad Software, San Diego, CA). *P* values<0.05 were considered statistically significant.

## Results

### Effects of NaB on MSC proliferation and differentiation

**Figure 1:**
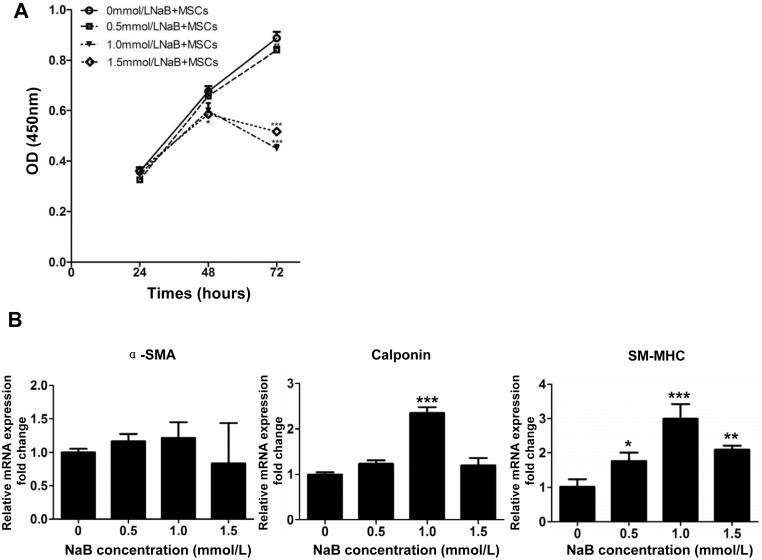
Effects of NaB on MSC proliferation and differentiation. (A) MSCs were harvested at 24, 48 and 72 h after the addtion of NaB and the proliferation of the treated MSCs was measured with a CCK-8 assay at the indicated time points. OD, optical density. *, *P*<0.05, ***, *P*<0.001 *vs*. the OD value of MSCs treated with 0 mmol/L NaB at each time points. (B) Relative expressions of the SMC specific genes (α-SMA, calponin and SM-MHC) in MSCs treated with the indicated concentration of NaB for 48 h. *β*-actin was used as the internal standard. *, *P*<0.05, **, *P*<0.01, ***, *P*<0.001 relative to 0 mmol/L NaB treated MSCs.

To examine the effects of NaB on MSC proliferation and differentiation, we treated MSCs with a series concentrations of NaB and observed the cell proliferation rate at different time points. As shown in **Fig. 1A**, MSC proliferation was not affected by 0.5 mmol/L NaB at any time point, as determined by a CCK-8 assay. However, MSC proliferation was significantly suppressed 48 hours after treatment with 1.0 and 1.5 mmol/L NaB.

**Figure 2:**
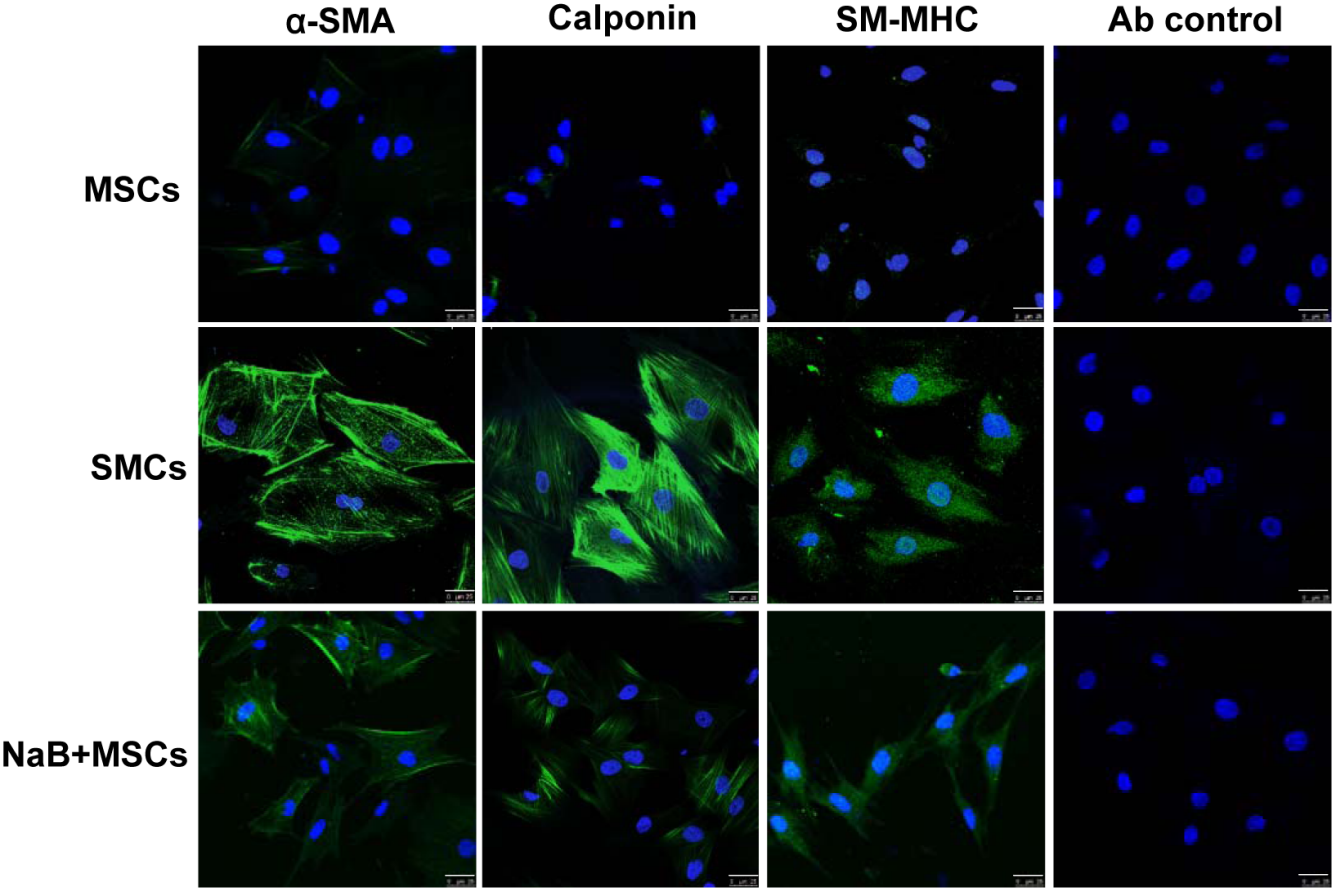
Immunofluorescence analysis of MSC specific protein expression in NaB-treated MSCs. MSCs were treated with 1 mmol/L NaB for 48 h, stained with FITC-conjugated anti-α-SMA, calponin or SM-MHC antibodies, and observed under a fluorescence microscope. The untreated MSCs were used as negative control and the primary SMCs were used as positive control. The isotype antibody was used as a background control. DAPI was used to stain the nuclei. Scale bar = 25 µm.

We then investigated the influence of NaB on the differentiation of MSCs to SMCs by determining the expression of the SMC specific genes α-SMA, calponin and SM-MHC in MSCs that were treated with different doses of NaB for 48 hours. The expression of calponin and SM-MHC increased after 48 hours of treatment with 1.0 and 1.5 mmol/L NaB, although NaB did not appear to influence the expression of α-SMA (**Fig. 1B**). To further investigate the expression of SMC specific genes at the protein level, we used an immunofluorescence assay to determine the levels of α-SMA, calponin and SM-MHC protein in MSCs 48 hours after treatment with 1.0 mmol/L NaB. The results indicated that NaB did indeed induce the expression of these SMCs specific genes at the protein level (**Fig. 2**).

### NaB exerts additional effects on MSC differentiation when co-cultured with SMCs

**Figure 3:**
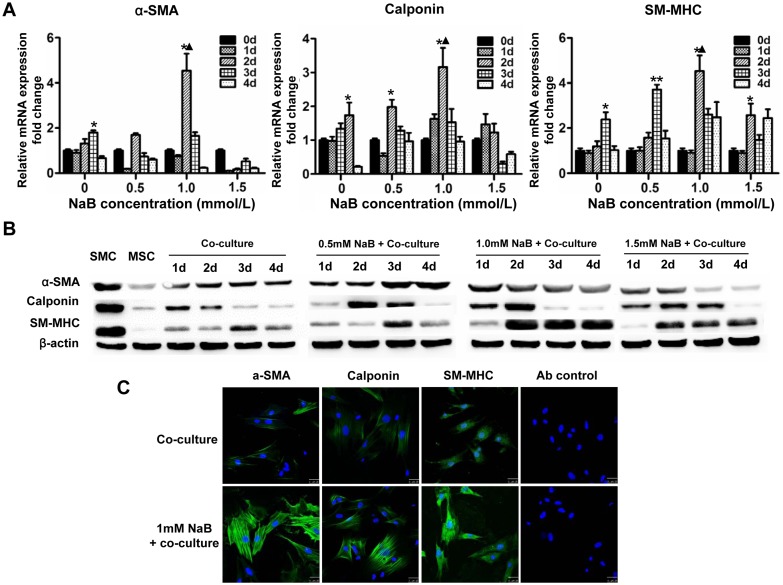
NaB induces SMC specific gene expression in MSCs co-cultured with SMCs. MSCs were co-cultured with SMCs in a transwell chamber with SMCs in the insert chamber and MSCs in the lower chamber. The MSCs were pretreated with 0, 0.5, 1.0 and 1.5 mM NaB before co-culturing. The MSCs in the co-culture system were harvested, and the expression of the SMC specific genes α-SMA, calponin and SM-MHC was determined by quantitative real-time RT-PCR (A) and Western blot (B). *, *P*<0.05, **, *P*<0.01 *vs*. all other time points in the same NaB concentration group; ▴, *P*<0.01 *vs*. all other time points in all NaB concentration groups. (C) The co-cultured MSCs were stimulated with 1 mmol/L NaB for 48 h, stained with FITC-conjugated anti-α-SMA, calponin or SM-MHC antibodies, and observed under a fluorescence microscope. DAPI was used to stain the cell nuclei. The isotype antibody was used as a background control. Scale bar = 25 µm.

Liao et al. reported that the co-seeding of bone marrow MSCs and SMCs into an acellular bladder matrix could successfully generate a tissue-engineered tubular graft for ureteral reconstruction in a rabbit model [5]. These results suggest that SMCs can direct MSC differentiation into SMCs. Therefore, we explored whether NaB could further promote the differentiation of MSCs to SMCs, which would support the potential clinical application of MSCs for related diseases. We first examined whether SMCs could assist the differentiation of MSCs into SMCs *in vitro* by co-culturing MSCs with SMCs in a transwell chamber system. The results of this assay indicated that the mRNA levels of the SMC specific genes α-SMA, calponin and SM-MHC in MSCs peaked after 3 days of co-culture with SMCs (**Fig. 3A**). Furthermore, the expression levels of these genes increased almost 2 fold in MSCs pretreated with 1.0 mM NaB before SMC co-culturing compared with MSCs without NaB treatment. Noticeably, the peak level for each target gene was reached after 2 days of co-culturing when the cells were pretreated with 1.0 mM NaB (**Fig. 3A**).

We next used a Western blot to investigate whether NaB could increase the protein level of MSC specific genes in the above co-culture system. As shown in **Fig. 3B**, the level of α-SMA protein in MSCs co-cultured with SMCs increased and peaked at 3 days of co-culturing; however, pre-treatment with 1.0 mM NaB caused α-SMA protein expression to peak at 1 day of co-culturing. Although NaB significantly enhanced calponin protein expression, NaB did not influence the time at which the calponin protein level peaked. In addition, while the level of SM-MHC protein in MSCs co-cultured with SMCs peaked after 3 days of co-culture, 1 mM NaB markedly increased the expression of SM-MHC protein and shifted the expression peak to 2 days of co-culturing. We finally used an immunofluorescence assay to confirm the effects of NaB on SMC specific gene expression. The results indicated that 1 mM NaB remarkably increased the expression of SMC specific genes after 2 days of co-culturing (**Fig. 3C**).

### NaB improves SMC specific genes expression by increasing H3K9^ace^ and H4^ace^ in the target genes

**Figure 4:**
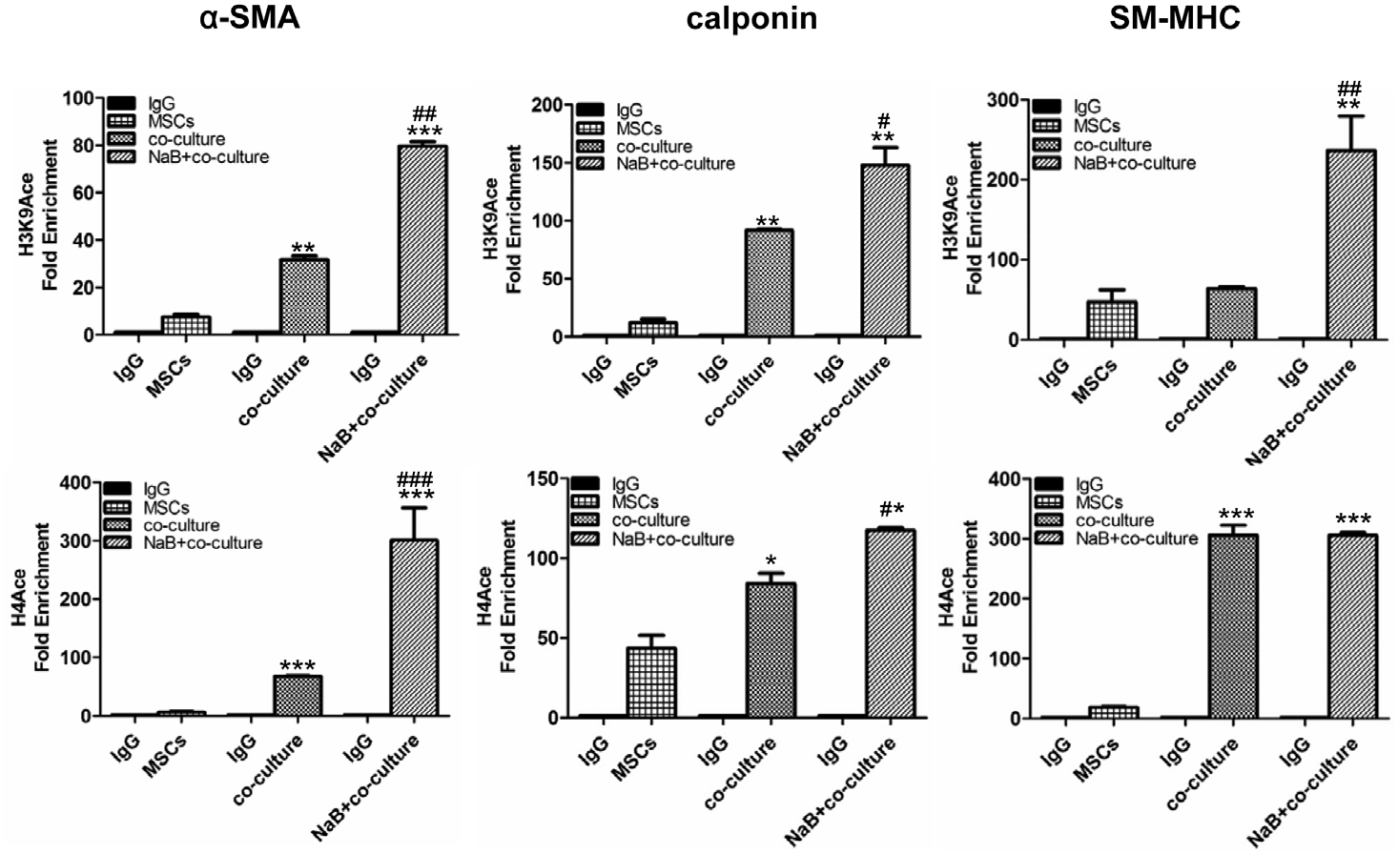
Histone acetylation modifications in co-cultured MSCs treated with NaB. MSCs were co-cultured with SMCs in a transwell chamber for 48 h. The MSCs were pretreated with 1.0 mM NaB before co-culturing. The MSCs in the co-culture system were harvested, and the genomic DNA was isolated and sonicated for a ChIP assay with antibodies against acetyl-histone H3K9, acetyl-histone H4 and normal rat IgG. The specific DNA fragments retrieved in the pull-down were further used for qPCR assays. The qPCR primers were designed to target the promoter of each gene. Values were given as folds of enrichment relative to the IgG control. Data are expressed as the mean ± SD of three biological replicates.*, *P*<0.05, **P<0.01, ***P<0.001 compared to the untreated MSCs. ^#^, *P*<0.05, ^##^ *P*<0.01, ^###^ *P*<0.001 compared to the co-cultured MSCs.

The acetylation of H3 and H4 has been reported to play an important role in the expression of SMCs specific genes [6], [7]. Therefore, we investigated whether NaB promoted MSC differentiation, which depends on the expression of SMC specific genes in MSCs. The expression of these genes is regulated by the acetylation of the promoter regions of these specific genes. To this end, we used ChIP-qPCR to determine the histone modifications within the promoters of these genes in differentiated MSCs that were treated with 1 mM NaB. The results of this assay showed that the acetylation of H3K9 and H4 at the promoters of the α-SMA and calponin genes increased significantly when MSCs were co-cultured with SMCs (**Fig. 4**). NaB further enhanced the acetylation of these genes. However, NaB increased the level of H3K9^ace^ at the SM-MHC promoter but did not influence the level of H4^ace^ in this gene (**Fig. 4**), which might reflect the complex regulation mechanisms of epigenetics.

### NaB inhibits the expression and recruitment of HDAC2 to SMCs specific genes in MSCs

**Figure 5:**
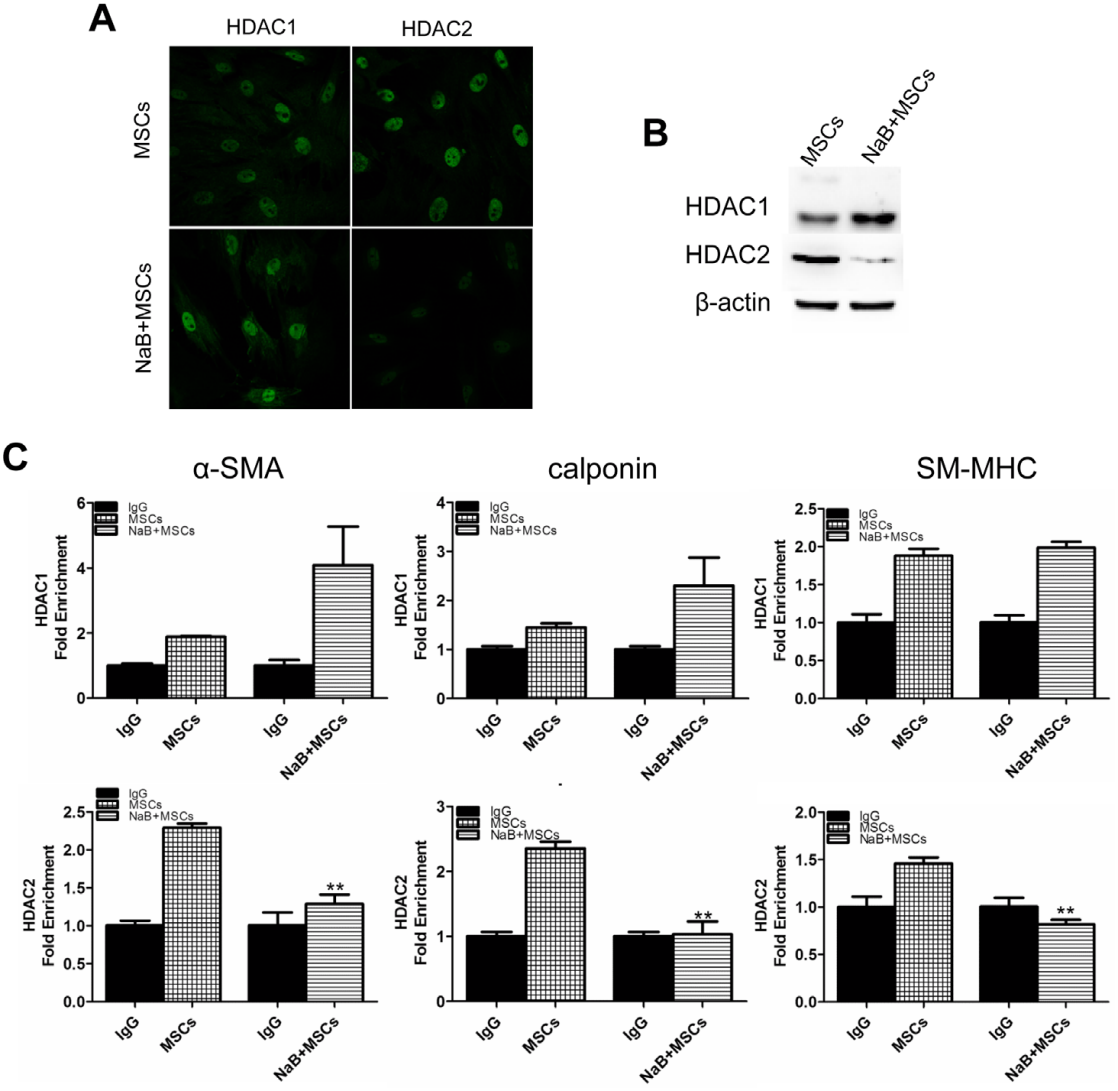
Effects of NaB on HDAC1/2 expression and recruitment in MSCs. (A) Immunofluorescence analysis of HDAC1 and HDAC2 expression in MSCs treated with NaB (1 mM for 48 h). (B) Western blot assay to determine the expression of HDAC1 and HDAC2 in MSCs treated with 1 mM NaB for 48 h. (C) ChIP-qPCR assay to determine the recruitment of HDAC1 and HDAC2 to the α-SMA, calponin and SM-MHC promoters in MSCs treated with 1 mM NaB for 48 h. The ChIP assay was conducted with ChIP grade anti-HDAC1, HDAC2 and normal rat IgG antibodies, which were incubated with the sonicated supernatants of MSCs treated with 1 mM NaB. The isolated DNA fragments were analyzed by qPCR to determine the presence of the promoter regions of the α-SMA, calponin and SM-MHC genes. Values were given as fold changes normalized with normal rat IgG control. **, *P*<0.01 compared to the untreated MSCs.

Histone acetylation status is regulated by the balance of histone acetyltransferases (HATs) and HDACs [9], and HDACs such as HDAC1 and HDAC2 are known to be involved in SMC specific gene expression [11], [12]. Because NaB inhibits HDACs, it is thus reasonable to deduce that NaB enhances MSC differentiation to SMCs by inhibiting the enzymatic capacity of HDACs. However, it has also been reported that the HDAC inhibitor trichostatin A (TSA) not only blocks HDAC activity but also elevates the expression of HDACs [18]. Therefore, in this study, we investigated whether NaB has also such effects on HDAC expression. We first used an immunofluorescence assay to examine HDAC1 and HDAC2 expression in MSCs treated with NaB. As shown in **Fig. 5A**, the HDAC1 and HDAC2 proteins were expressed in the nuclei of MSCs. Treatment with 1 mM NaB increased HDAC1 expression slightly, but significantly down-regulated HDAC2 expression. A Western-blot assay further confirmed these results (**Fig. 5B**). We next investigated whether the altered HDAC1/2 expression influenced SMC specific gene expression in MSCs by regulating the recruitment of HDAC1/2 to the promoter region of the target genes with a ChIP-qPCR assay. Although HDAC1 expression was slightly up-regulated by NaB, treatment with NaB did not significantly change HDAC1 recruitment to the α-SMA, calponin and SM-MHC promoters. However, NaB treatment remarkably reduced the recruitment of HDAC2 to the promoters of these genes (**Fig. 5C**), a finding that was in accordance with the NaB-induced decrease in HDAC2 expression (**Fig. 5A and 5B**).

### Differentiated MSCs show agonist-induced calcium transients

**Figure 6:**
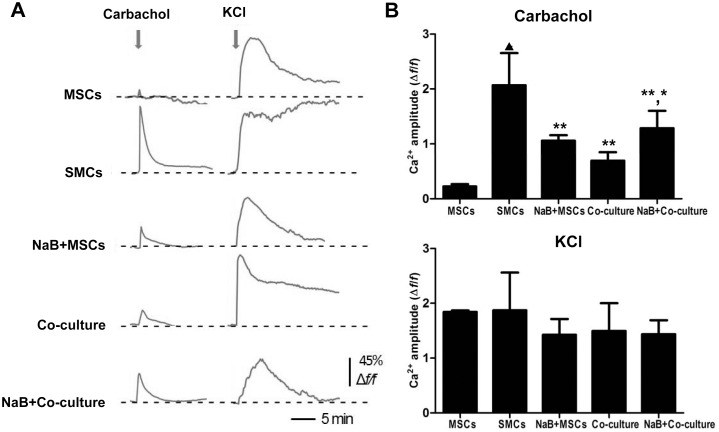
Measurement of intracellular calcium levels. (A) The traces show the carbachol- (10 mM) and KCl (100 mM)-induced Ca^2+^ response in MSCs, SMCs, MSCs treated with 1 mM NaB for 48 h, and NaB-pretreated (1 mM for 48 h) MSCs co-cultured with SMCs for 3 d. F/F0 denotes the relative change in Fluo-8 intensity, F0 is the baseline average and F is the absolute fluorescence value in an area of interest during treatment. Forty-five percent means that 45 of one hundred cells exhibited a response. (B) The amplitude of the Ca^2+^ peak in response to carbachol (10 mM) or KCl (100 mM) in MSCs, SMCs, MSCs treated with NaB, and NaB-pretreated MSCs co-cultured with SMCs. *P<0.05 *vs.* MSCs, NaB+MSCs and co-cultured MSCs; **P<0.01 *vs.* MSCs; ^▴^ *P*<0.01 *vs.* all other gourps.

Mature SMCs have the ability to contract, a characteristic that is controlled by the Ca^2+^ and Rho kinase signaling pathways [19]. Phasic SMCs (which occur in the vas deferens, uterus and bladder) rely on membrane depolarization to drive Ca^2+^ influx across the plasma membrane [19]. To investigate whether the differentiated MSCs generated in this study have the ability to contract, we measured Ca^2+^ influx in these cells. MSCs, SMCs and co-cultured MSCs pretreated with NaB were loaded with Fluo-8 AM to determine their response to 10 mM carbachol. The depolarization of the cells was recorded by fluorescence microscopy. As shown in **Fig. 6**, the intracellular Ca^2+^ levels in NaB-treated MSCs, co-cultured MSCs and NaB-treated co-cultured MSCs were higher than the intracellular Ca^2+^ levels in the untreated MSCs. Of the treatments, the NaB-treated co-cultured MSCs exhibited the greatest Ca^2+^ influx; however, this value was still lower than the Ca^2+^ influx in the SMCs, the positive control in this study. The cells in all of the groups were depolarized by a high concentration of KCl (**Fig. 6**), indicating the viability of the cells.

## Discussion

The clinical applications of tissue engineering are limited by the source of stem cells [20], [21]. MSCs are an ideal seed cell due to their potential to differentiate into a variety of cells [21]. Although a report has indicated that MSCs can be induced to differentiate into SMCs via co-culturing with SMCs [22], our results indicate that NaB significantly enhances MSC differentiation into SMCs in such a co-culture system. Further investigation demonstrated that NaB markedly decreased the expression of HDAC2 and its recruitment to SMC specific genes in MSCs, which further induced the increased acetylation of H3K9 and H4 and the subsequent increase in the expression of the SMC specific genes α-SMA, calponin and SM-MHC. Finally and importantly, the NaB-induced differentiated MSCs showed more sensitive depolarization in response to carbachol stimulation, as indicated by a higher intracellular calcium level, verifying the functionality of NaB-induced differentiated MSCs.

Currently, the application of stem cell transplantation is hindered by the low differentiation efficiency of stem cells [20], [21]. Many labs have devoted themselves to promoting the differentiation of stem cells *in vitro* or *in vivo* [20], [21]. Recently, HDAC inhibitors have received more attention for arresting the cell cycle of stem cells, inducing cell differentiation, and/or promoting cell apoptosis [23], [24]. NaB is an HDAC inhibitor that has been proven to inhibit the proliferation of stem cells [25], [26]. We also found that treatment with 1 mmol/L NaB for 48 h could suppress MSC proliferation. In addition, previous studies have shown that NaB can induce stem cells to express cell-type specific markers [13], [27]. Our results are the first to show that treating rat MSCs with NaB can markedly increase the expression of α-SMA, calponin and SM-MHC, which are the early, middle and late SMC marker genes. Furthermore, NaB significantly enhanced the expression of these SMC specific genes in MSCs co-cultured with SMCs compared with MSCs co-cultured with SMCs in the absence of NaB. Our findings indicate that NaB is an efficient candidate to promote MSC differentiation to SMCs.

Histone acetylation status is regulated by the balance of histone acetyltransferases (HATs) and HDACs [9]. HDAC1 and HDAC2 are class I HDACs that have 85% sequence similarity and are usually co-expressed to repress gene transcription [9]. HDACs such as HDAC1 and HDAC2 are known to be involved in SMC specific gene expression [11], [12] [28]. As an inhibitor of HDACs, NaB should enhance MSC differentiation to SMCs by inhibiting the enzymatic capacity of HDACs. However, the HDACs inhibitor trichostatin A (TSA) not only blocks HDACs activity but also elevates the expression of HDAC proteins [18]. In this study, the immunofluorescence staining and Western blot assays showed that NaB elevated HDAC1 expression slightly but significantly down-regulated HDAC2 expression. More importantly, the ChIP-qPCR assay demonstrated that while HDAC1 expression was slightly up-regulated by NaB, HDAC1 recruitment to the α-SMA, calponin and SM-MHC promoters was not significantly changed. However, NaB treatment remarkably reduced HDAC2 recruitment to the promoters of these genes. These epigenetic changes in NaB-treated MSCs caused the increased expression of SMC specific genes, reflecting the differentiation of MSCs to SMCs.

Previous studies have shown that NaB increases gene expression by promoting the hyperacetylation of the histones at target loci [29], [30]. Several studies suggest that histone modifications play an important role in SMC differentiation in vitro [31], [32]. Recently, several reports demonstrated that specific histone modifications are acquired at the promoters of SMC genes in the transition from ECSs to SMCs [33]. Acetylated H3 and H4 are enriched at the α-SMA gene in SMCs, whereas stem cell lines possess low acetylation levels [33]. Consistent with these findings, we also found that the levels of H3K9^ace^ and H4^ace^ at SMC specific genes were higher in the differentiated MSCs than undifferentiated MSCs, which means that the acetylation of H3K9 and H4 initiates gene expression during the differentiation of MSCs to SMCs. In addition, the levels of H4^ace^ and H3K9^ace^ were much higher in differentiated MSCs pretreated with NaB, which is consistent with the view that NaB can directly increase H4 acetylation [23]. These data suggest that NaB increases the levels of H3K9^ace^ and H4^ace^ to activate SMC specific gene expression during the differentiation of MSCs. Further research is necessary to identify how H3K9^ace^ cooperates with H4^ace^ to increase SMC specific genes expression during NaB-induced MSC differentiation.

Functional SMCs contract efficiently in response to depolarizing agonists such as carbachol [34], and previous studies have shown that ESC-derived SMCs exhibit agonist-induced calcium transients [35]. Our results demonstrated that the intracellular Ca^2+^ level of differentiated MSCs increased significantly upon carbachol stimulation, indicating that NaB-induced differentiated MSCs displayed the appropriate excitation and contractile responses to a depolarizing agonist.

In conclusion, NaB effectively promotes MSC differentiation into SMCs. The mechanisms might include the NaB-mediated decrease of HDAC2 expression and its recruitment to SMC specific genes in MSCs, which further induces high levels of H3K9^ace^ and H4^ace^ and the subsequent expression of target genes. This study provides a potential strategy to promote MSC differentiation into SMCs that could potentially be applied in clinical tissue engineering and cell transplantation.

## Supporting Information

S1 Fig Flow cytometry assay to identify MSCs. Bone marrow MSCs were aseptically isolated from the femurs and tibias of rats, and the third or fourth passage cells were incubated with FITC-labeled monoclonal antibodies against CD29, CD31, CD34, and CD45 for 30 min at 4°C and analyzed by flow cytometry to determine the surface marker expression of the MSCs.
(TIF)
