# Supplementary figures and images for: Sodium Butyrate Promotes the Differentiation of Rat Bone Marrow Mesenchymal Stem Cells to Smooth Muscle Cells through Histone Acetylation
Source: PLoS One. 2014 Dec 30;9(12):e116183. doi: 10.1371/journal.pone.0116183 (PMC4280132; doi:10.1371/journal.pone.0116183)

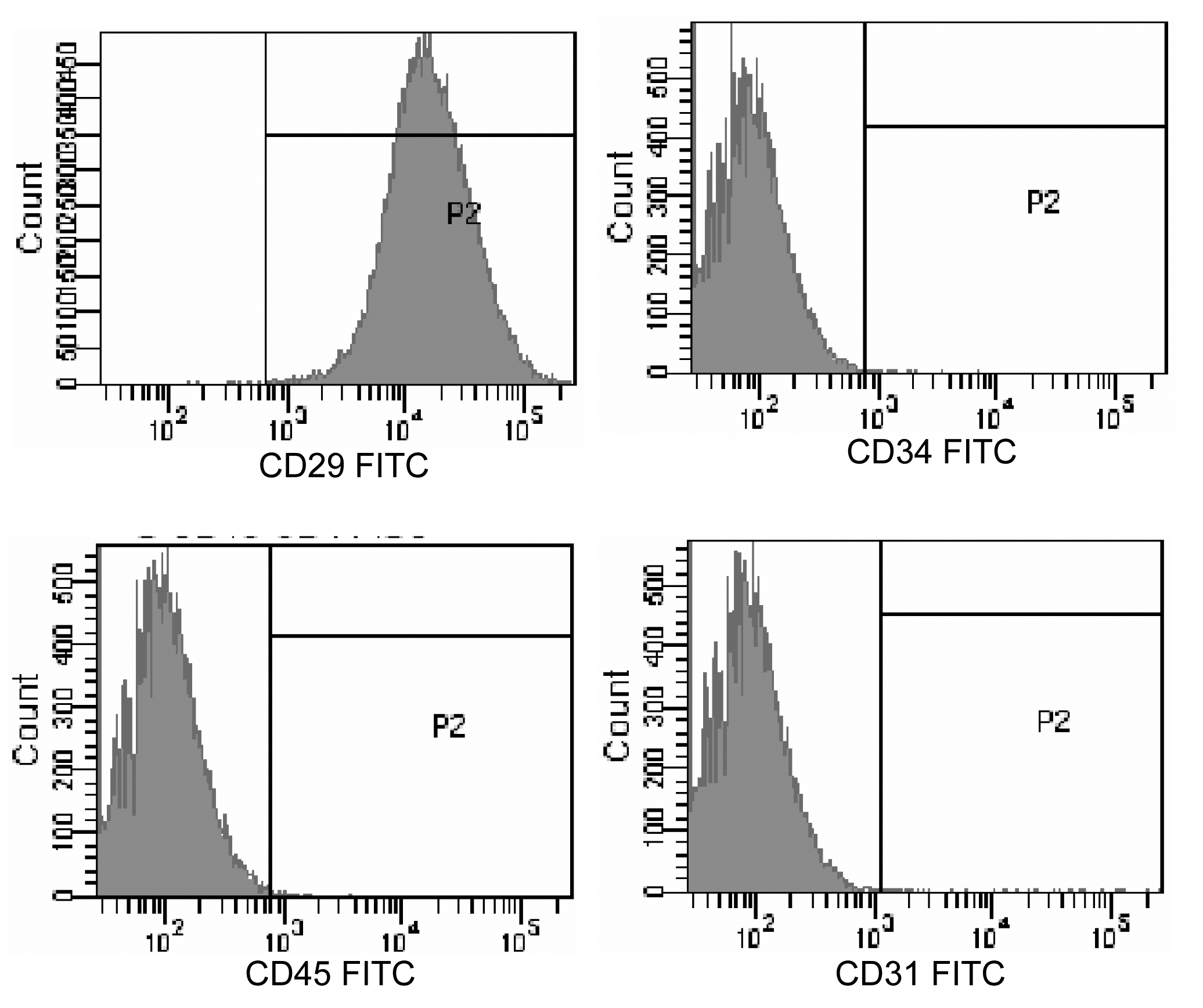

Supplement: S1 Fig — Flow cytometry assay to identify MSCs. Bone marrow MSCs were aseptically isolated from the femurs and tibias of rats, and the third or fourth passage cells were incubated with FITC-labeled monoclonal antibodies against CD29, CD31, CD34, and CD45 for 30 min at 4°C and analyzed by flow cytometry to determine the surface marker expression of the MSCs. (TIF) [file pone.0116183.s001.tif]
